# Supplementary material for: HIVprotI: an integrated web based platform for prediction and design of HIV proteins inhibitors
Source: J Cheminform. 2018 Mar 9;10:12. doi: 10.1186/s13321-018-0266-y (PMC5845081; doi:10.1186/s13321-018-0266-y)
Supplement: Supplementary file 2 — Additional file 2. Source code of HIVProtI web server. [file 13321_2018_266_MOESM2_ESM.zip › HIVprotI_Source-code/hivprotI/weka-3-6-6/doc/weka/gui/beans/MetaBean.html]

MetaBean


---


|  |  |  |
| --- | --- | --- |
| **Overview** \| **Package** \| **Class** \| **Tree** \| **Deprecated** \| **Index** \| **Help** \| **Weka's home** | |  |
| **PREV CLASS**   **NEXT CLASS** | **FRAMES**    **NO FRAMES**     **All Classes** |  |
| SUMMARY: NESTED \| FIELD \| CONSTR \| METHOD | DETAIL: FIELD \| CONSTR \| METHOD |  |


---


## weka.gui.beans Class MetaBean

```
java.lang.Object
  java.awt.Component
      java.awt.Container
          javax.swing.JComponent
              javax.swing.JPanel
                  weka.gui.beans.MetaBean
```

**All Implemented Interfaces:**: java.awt.image.ImageObserver, java.awt.MenuContainer, java.io.Serializable, javax.accessibility.Accessible, BeanCommon, EventConstraints, UserRequestAcceptor, Visible

---

``` public class MetaBean extends javax.swing.JPanel implements BeanCommon, Visible, EventConstraints, java.io.Serializable, UserRequestAcceptor ```

A meta bean that encapsulates several other regular beans, useful for
grouping large KnowledgeFlows.

**Version:**
:   $Revision: 6017 $

**Author:**
:   Mark Hall (mhall at cs dot waikato dot ac dot nz)

**See Also:**: Serialized Form

---

**Nested Class Summary**

**Nested classes/interfaces inherited from class javax.swing.JComponent**

`javax.swing.JComponent.AccessibleJComponent`

**Nested classes/interfaces inherited from class java.awt.Component**

`java.awt.Component.BaselineResizeBehavior`


**Field Summary**

**Fields inherited from class javax.swing.JComponent**

`TOOL_TIP_TEXT_KEY, UNDEFINED_CONDITION, WHEN_ANCESTOR_OF_FOCUSED_COMPONENT, WHEN_FOCUSED, WHEN_IN_FOCUSED_WINDOW`

**Fields inherited from class java.awt.Component**

`BOTTOM_ALIGNMENT, CENTER_ALIGNMENT, LEFT_ALIGNMENT, RIGHT_ALIGNMENT, TOP_ALIGNMENT`

**Fields inherited from interface java.awt.image.ImageObserver**

`ABORT, ALLBITS, ERROR, FRAMEBITS, HEIGHT, PROPERTIES, SOMEBITS, WIDTH`


**Constructor Summary**

`MetaBean()`


| **Method Summary** | |
| --- | --- |
| `void` | `addPropertyChangeListenersSubFlow(java.beans.PropertyChangeListener pcl)` |
| `boolean` | `canAcceptConnection(java.lang.Class listenerClass)`             Checks to see if any of the inputs to this group implements the supplied listener class |
| `boolean` | `connectionAllowed(java.beans.EventSetDescriptor esd)`             Returns true if, at this time, the object will accept a connection with respect to the supplied EventSetDescriptor |
| `boolean` | `connectionAllowed(java.lang.String eventName)`             Returns true if, at this time, the object will accept a connection via the named event |
| `void` | `connectionNotification(java.lang.String eventName, java.lang.Object source)`             Notify this object that it has been registered as a listener with a source with respect to the named event. |
| `void` | `disconnectionNotification(java.lang.String eventName, java.lang.Object source)`             Notify this object that it has been deregistered as a listener with a source with respect to the supplied event name. |
| `java.util.Enumeration` | `enumerateRequests()`             Return an enumeration of requests that can be made by the user |
| `boolean` | `eventGeneratable(java.beans.EventSetDescriptor esd)`             Returns true, if at the current time, the event described by the supplied event descriptor could be generated. |
| `boolean` | `eventGeneratable(java.lang.String eventName)`             Returns true, if at the current time, the named event could be generated. |
| `java.util.Vector` | `getAssociatedConnections()` |
| `java.util.Vector` | `getBeanInfoInputs()` |
| `java.util.Vector` | `getBeanInfoOutputs()` |
| `java.util.Vector` | `getBeanInfoSubFlow()` |
| `java.util.Vector` | `getBeansInInputs()`             Return all the beans in the inputs |
| `java.util.Vector` | `getBeansInOutputs()`             Return all the beans in the outputs |
| `java.util.Vector` | `getBeansInSubFlow()`             Return all the beans in the sub flow |
| `java.lang.String` | `getCustomName()`             Get the custom (descriptive) name for this bean (if one has been set) |
| `java.util.Vector` | `getInputs()` |
| `java.util.Vector` | `getOriginalCoords()`             returns the vector containing the original coordinates (instances of class Point) for the inputs |
| `java.util.Vector` | `getOutputs()` |
| `java.util.Vector` | `getSubFlow()` |
| `java.util.Vector` | `getSuitableTargets(java.beans.EventSetDescriptor esd)`             Return a list of input beans capable of receiving the supplied event |
| `BeanVisual` | `getVisual()`             Gets the visual appearance of this wrapper bean |
| `boolean` | `inputsContains(BeanInstance toCheck)` |
| `boolean` | `isBusy()`             Returns true if. |
| `boolean` | `outputsContains(BeanInstance toCheck)` |
| `void` | `performRequest(java.lang.String request)`             Perform a particular request |
| `void` | `removePropertyChangeListenersSubFlow(java.beans.PropertyChangeListener pcl)` |
| `void` | `restoreBeans()` |
| `void` | `setAssociatedConnections(java.util.Vector ac)` |
| `void` | `setCustomName(java.lang.String name)`             Set a custom (descriptive) name for this bean |
| `void` | `setInputs(java.util.Vector inputs)` |
| `void` | `setLog(Logger logger)`             Set a logger |
| `void` | `setOriginalCoords(java.util.Vector value)`             sets the vector containing the original coordinates (instances of class Point) for the inputs |
| `void` | `setOutputs(java.util.Vector outputs)` |
| `void` | `setSubFlow(java.util.Vector sub)` |
| `void` | `setSubFlowPreview(javax.swing.ImageIcon sfp)` |
| `void` | `setVisual(BeanVisual newVisual)`             Sets the visual appearance of this wrapper bean |
| `void` | `shiftBeans(BeanInstance toShiftTo, boolean save)`             Move coords of all inputs and outputs of this meta bean to the coords of the supplied BeanInstance. |
| `void` | `stop()`             Stop all encapsulated beans |
| `boolean` | `subFlowContains(BeanInstance toCheck)` |
| `void` | `useDefaultVisual()`             Use the default visual appearance for this bean |

**Methods inherited from class javax.swing.JPanel**

`getAccessibleContext, getUI, getUIClassID, setUI, updateUI`

**Methods inherited from class javax.swing.JComponent**

`addAncestorListener, addNotify, addVetoableChangeListener, computeVisibleRect, contains, createToolTip, disable, enable, firePropertyChange, firePropertyChange, firePropertyChange, getActionForKeyStroke, getActionMap, getAlignmentX, getAlignmentY, getAncestorListeners, getAutoscrolls, getBaseline, getBaselineResizeBehavior, getBorder, getBounds, getClientProperty, getComponentPopupMenu, getConditionForKeyStroke, getDebugGraphicsOptions, getDefaultLocale, getFontMetrics, getGraphics, getHeight, getInheritsPopupMenu, getInputMap, getInputMap, getInputVerifier, getInsets, getInsets, getListeners, getLocation, getMaximumSize, getMinimumSize, getNextFocusableComponent, getPopupLocation, getPreferredSize, getRegisteredKeyStrokes, getRootPane, getSize, getToolTipLocation, getToolTipText, getToolTipText, getTopLevelAncestor, getTransferHandler, getVerifyInputWhenFocusTarget, getVetoableChangeListeners, getVisibleRect, getWidth, getX, getY, grabFocus, isDoubleBuffered, isLightweightComponent, isManagingFocus, isOpaque, isOptimizedDrawingEnabled, isPaintingForPrint, isPaintingTile, isRequestFocusEnabled, isValidateRoot, paint, paintImmediately, paintImmediately, print, printAll, putClientProperty, registerKeyboardAction, registerKeyboardAction, removeAncestorListener, removeNotify, removeVetoableChangeListener, repaint, repaint, requestDefaultFocus, requestFocus, requestFocus, requestFocusInWindow, resetKeyboardActions, reshape, revalidate, scrollRectToVisible, setActionMap, setAlignmentX, setAlignmentY, setAutoscrolls, setBackground, setBorder, setComponentPopupMenu, setDebugGraphicsOptions, setDefaultLocale, setDoubleBuffered, setEnabled, setFocusTraversalKeys, setFont, setForeground, setInheritsPopupMenu, setInputMap, setInputVerifier, setMaximumSize, setMinimumSize, setNextFocusableComponent, setOpaque, setPreferredSize, setRequestFocusEnabled, setToolTipText, setTransferHandler, setVerifyInputWhenFocusTarget, setVisible, unregisterKeyboardAction, update`

**Methods inherited from class java.awt.Container**

`add, add, add, add, add, addContainerListener, addPropertyChangeListener, addPropertyChangeListener, applyComponentOrientation, areFocusTraversalKeysSet, countComponents, deliverEvent, doLayout, findComponentAt, findComponentAt, getComponent, getComponentAt, getComponentAt, getComponentCount, getComponents, getComponentZOrder, getContainerListeners, getFocusTraversalKeys, getFocusTraversalPolicy, getLayout, getMousePosition, insets, invalidate, isAncestorOf, isFocusCycleRoot, isFocusCycleRoot, isFocusTraversalPolicyProvider, isFocusTraversalPolicySet, layout, list, list, locate, minimumSize, paintComponents, preferredSize, printComponents, remove, remove, removeAll, removeContainerListener, setComponentZOrder, setFocusCycleRoot, setFocusTraversalPolicy, setFocusTraversalPolicyProvider, setLayout, transferFocusBackward, transferFocusDownCycle, validate`

**Methods inherited from class java.awt.Component**

`action, add, addComponentListener, addFocusListener, addHierarchyBoundsListener, addHierarchyListener, addInputMethodListener, addKeyListener, addMouseListener, addMouseMotionListener, addMouseWheelListener, bounds, checkImage, checkImage, contains, createImage, createImage, createVolatileImage, createVolatileImage, dispatchEvent, enable, enableInputMethods, firePropertyChange, firePropertyChange, firePropertyChange, firePropertyChange, firePropertyChange, getBackground, getBounds, getColorModel, getComponentListeners, getComponentOrientation, getCursor, getDropTarget, getFocusCycleRootAncestor, getFocusListeners, getFocusTraversalKeysEnabled, getFont, getForeground, getGraphicsConfiguration, getHierarchyBoundsListeners, getHierarchyListeners, getIgnoreRepaint, getInputContext, getInputMethodListeners, getInputMethodRequests, getKeyListeners, getLocale, getLocation, getLocationOnScreen, getMouseListeners, getMouseMotionListeners, getMousePosition, getMouseWheelListeners, getName, getParent, getPeer, getPropertyChangeListeners, getPropertyChangeListeners, getSize, getToolkit, getTreeLock, gotFocus, handleEvent, hasFocus, hide, imageUpdate, inside, isBackgroundSet, isCursorSet, isDisplayable, isEnabled, isFocusable, isFocusOwner, isFocusTraversable, isFontSet, isForegroundSet, isLightweight, isMaximumSizeSet, isMinimumSizeSet, isPreferredSizeSet, isShowing, isValid, isVisible, keyDown, keyUp, list, list, list, location, lostFocus, mouseDown, mouseDrag, mouseEnter, mouseExit, mouseMove, mouseUp, move, nextFocus, paintAll, postEvent, prepareImage, prepareImage, remove, removeComponentListener, removeFocusListener, removeHierarchyBoundsListener, removeHierarchyListener, removeInputMethodListener, removeKeyListener, removeMouseListener, removeMouseMotionListener, removeMouseWheelListener, removePropertyChangeListener, removePropertyChangeListener, repaint, repaint, repaint, resize, resize, setBounds, setBounds, setComponentOrientation, setCursor, setDropTarget, setFocusable, setFocusTraversalKeysEnabled, setIgnoreRepaint, setLocale, setLocation, setLocation, setName, setSize, setSize, show, show, size, toString, transferFocus, transferFocusUpCycle`

**Methods inherited from class java.lang.Object**

`equals, getClass, hashCode, notify, notifyAll, wait, wait, wait`

**Constructor Detail**

### MetaBean

```
public MetaBean()
```


**Method Detail**

### setCustomName

```
public void setCustomName(java.lang.String name)
```

:   Set a custom (descriptive) name for this bean

    :   **Specified by:**: `setCustomName` in interface `BeanCommon`
    :   **Parameters:**: `name` - the name to use

---


### getCustomName

```
public java.lang.String getCustomName()
```

:   Get the custom (descriptive) name for this bean (if one has been set)

    :   **Specified by:**: `getCustomName` in interface `BeanCommon`
    :   **Returns:**: the custom name (or the default name)

---


### setAssociatedConnections

```
public void setAssociatedConnections(java.util.Vector ac)
```

---


### getAssociatedConnections

```
public java.util.Vector getAssociatedConnections()
```

---


### setSubFlow

```
public void setSubFlow(java.util.Vector sub)
```

---


### getSubFlow

```
public java.util.Vector getSubFlow()
```

---


### setInputs

```
public void setInputs(java.util.Vector inputs)
```

---


### getInputs

```
public java.util.Vector getInputs()
```

---


### setOutputs

```
public void setOutputs(java.util.Vector outputs)
```

---


### getOutputs

```
public java.util.Vector getOutputs()
```

---


### subFlowContains

```
public boolean subFlowContains(BeanInstance toCheck)
```

---


### inputsContains

```
public boolean inputsContains(BeanInstance toCheck)
```

---


### outputsContains

```
public boolean outputsContains(BeanInstance toCheck)
```

---


### getBeansInSubFlow

```
public java.util.Vector getBeansInSubFlow()
```

:   Return all the beans in the sub flow

    :   **Returns:**: a Vector of all the beans in the sub flow

---


### getBeansInInputs

```
public java.util.Vector getBeansInInputs()
```

:   Return all the beans in the inputs

    :   **Returns:**: a Vector of all the beans in the inputs

---


### getBeansInOutputs

```
public java.util.Vector getBeansInOutputs()
```

:   Return all the beans in the outputs

    :   **Returns:**: a Vector of all the beans in the outputs

---


### getBeanInfoSubFlow

```
public java.util.Vector getBeanInfoSubFlow()
```

---


### getBeanInfoInputs

```
public java.util.Vector getBeanInfoInputs()
```

---


### getBeanInfoOutputs

```
public java.util.Vector getBeanInfoOutputs()
```

---


### getOriginalCoords

```
public java.util.Vector getOriginalCoords()
```

:   returns the vector containing the original coordinates (instances of class
    Point) for the inputs

    :   **Returns:**: the containing the coord Points of the original inputs

---


### setOriginalCoords

```
public void setOriginalCoords(java.util.Vector value)
```

:   sets the vector containing the original coordinates (instances of class
    Point) for the inputs

    :   **Parameters:**: `value` - the vector containing the points of the coords of the original inputs

---


### shiftBeans

```
public void shiftBeans(BeanInstance toShiftTo,
                       boolean save)
```

:   Move coords of all inputs and outputs of this meta bean
    to the coords of the supplied BeanInstance. Typically
    the supplied BeanInstance is the BeanInstance that encapsulates
    this meta bean; the result in this case is that all inputs
    and outputs are shifted so that their coords coincide with
    the meta bean and all connections to them appear (visually) to
    go to/from the meta bean.

    :   **Parameters:**: `toShiftTo` - the BeanInstance whos coordinates will be used.: `save` - true if coordinates are to be saved.

---


### restoreBeans

```
public void restoreBeans()
```

---


### eventGeneratable

```
public boolean eventGeneratable(java.beans.EventSetDescriptor esd)
```

:   Returns true, if at the current time, the event described by the
    supplied event descriptor could be generated.

    :   **Parameters:**: `esd` - an `EventSetDescriptor` value **Returns:**: a `boolean` value

---


### eventGeneratable

```
public boolean eventGeneratable(java.lang.String eventName)
```

:   Returns true, if at the current time, the named event could
    be generated. Assumes that the supplied event name is
    an event that could be generated by this bean

    :   **Specified by:**: `eventGeneratable` in interface `EventConstraints`
    :   **Parameters:**: `eventName` - the name of the event in question **Returns:**: true if the named event could be generated at this point in time

---


### connectionAllowed

```
public boolean connectionAllowed(java.beans.EventSetDescriptor esd)
```

:   Returns true if, at this time,
    the object will accept a connection with respect to the
    supplied EventSetDescriptor

    :   **Specified by:**: `connectionAllowed` in interface `BeanCommon`
    :   **Parameters:**: `esd` - the EventSetDescriptor **Returns:**: true if the object will accept a connection

---


### connectionAllowed

```
public boolean connectionAllowed(java.lang.String eventName)
```

:   **Description copied from interface: `BeanCommon`**
:   Returns true if, at this time,
    the object will accept a connection via the named event

    :   **Specified by:**: `connectionAllowed` in interface `BeanCommon`
    :   **Parameters:**: `eventName` - the name of the event **Returns:**: true if the object will accept a connection

---


### connectionNotification

```
public void connectionNotification(java.lang.String eventName,
                                   java.lang.Object source)
```

:   Notify this object that it has been registered as a listener with
    a source with respect to the named event. This is just a dummy
    method in this class to satisfy the interface. Specific code
    in BeanConnection takes care of this method for MetaBeans

    :   **Specified by:**: `connectionNotification` in interface `BeanCommon`
    :   **Parameters:**: `eventName` - the event: `source` - the source with which this object has been registered as a listener

---


### disconnectionNotification

```
public void disconnectionNotification(java.lang.String eventName,
                                      java.lang.Object source)
```

:   Notify this object that it has been deregistered as a listener with
    a source with respect to the supplied event name. This is just a dummy
    method in this class to satisfy the interface. Specific code
    in BeanConnection takes care of this method for MetaBeans

    :   **Specified by:**: `disconnectionNotification` in interface `BeanCommon`
    :   **Parameters:**: `eventName` - the event: `source` - the source with which this object has been registered as a listener

---


### stop

```
public void stop()
```

:   Stop all encapsulated beans

    :   **Specified by:**: `stop` in interface `BeanCommon`

---


### isBusy

```
public boolean isBusy()
```

:   Returns true if. at this time, the bean is busy with some
    (i.e. perhaps a worker thread is performing some calculation).

    :   **Specified by:**: `isBusy` in interface `BeanCommon`
    :   **Returns:**: true if the bean is busy.

---


### setVisual

```
public void setVisual(BeanVisual newVisual)
```

:   Sets the visual appearance of this wrapper bean

    :   **Specified by:**: `setVisual` in interface `Visible`
    :   **Parameters:**: `newVisual` - a `BeanVisual` value

---


### getVisual

```
public BeanVisual getVisual()
```

:   Gets the visual appearance of this wrapper bean

    :   **Specified by:**: `getVisual` in interface `Visible`
    :   **Returns:**: a `BeanVisual` value

---


### useDefaultVisual

```
public void useDefaultVisual()
```

:   Use the default visual appearance for this bean

    :   **Specified by:**: `useDefaultVisual` in interface `Visible`

---


### enumerateRequests

```
public java.util.Enumeration enumerateRequests()
```

:   Return an enumeration of requests that can be made by the user

    :   **Specified by:**: `enumerateRequests` in interface `UserRequestAcceptor`
    :   **Returns:**: an `Enumeration` value

---


### setSubFlowPreview

```
public void setSubFlowPreview(javax.swing.ImageIcon sfp)
```

---


### performRequest

```
public void performRequest(java.lang.String request)
```

:   Perform a particular request

    :   **Specified by:**: `performRequest` in interface `UserRequestAcceptor`
    :   **Parameters:**: `request` - the request to perform **Throws:**: `java.lang.IllegalArgumentException` - if an error occurs

---


### setLog

```
public void setLog(Logger logger)
```

:   Set a logger

    :   **Specified by:**: `setLog` in interface `BeanCommon`
    :   **Parameters:**: `logger` - a `Logger` value

---


### removePropertyChangeListenersSubFlow

```
public void removePropertyChangeListenersSubFlow(java.beans.PropertyChangeListener pcl)
```

---


### addPropertyChangeListenersSubFlow

```
public void addPropertyChangeListenersSubFlow(java.beans.PropertyChangeListener pcl)
```

---


### canAcceptConnection

```
public boolean canAcceptConnection(java.lang.Class listenerClass)
```

:   Checks to see if any of the inputs to this group implements
    the supplied listener class

    :   **Parameters:**: `listenerClass` - the listener to check for

---


### getSuitableTargets

```
public java.util.Vector getSuitableTargets(java.beans.EventSetDescriptor esd)
```

:   Return a list of input beans capable of receiving the
    supplied event

    :   **Parameters:**: `esd` - the event in question **Returns:**: a vector of beans capable of handling the event


---


|  |  |  |
| --- | --- | --- |
| **Overview** \| **Package** \| **Class** \| **Tree** \| **Deprecated** \| **Index** \| **Help** \| **Weka's home** | |  |
| **PREV CLASS**   **NEXT CLASS** | **FRAMES**    **NO FRAMES**     **All Classes** |  |
| SUMMARY: NESTED \| FIELD \| CONSTR \| METHOD | DETAIL: FIELD \| CONSTR \| METHOD |  |


---
